# Electroacupuncture Inhibits Neuronal Autophagy and Apoptosis *via* the PI3K/AKT Pathway Following Ischemic Stroke

**DOI:** 10.3389/fncel.2020.00134

**Published:** 2020-05-15

**Authors:** Man-Man Wang, Min Zhang, Ya-Shuo Feng, Ying Xing, Zi-Xuan Tan, Wen-Bin Li, Fang Dong, Feng Zhang

**Affiliations:** ^1^Department of Rehabilitation Medicine, The Third Hospital of Hebei Medical University, Shijiazhuang, China; ^2^Department of Pathophysiology, Hebei Medical University, Shijiazhuang, China; ^3^Department of Clinical Laboratory Medicine, The Third Hospital of Hebei Medical University, Shijiazhuang, China; ^4^Hebei Provincial Orthopedic Biomechanics Key Laboratory, The Third Hospital of Hebei Medical University, Shijiazhuang, China

**Keywords:** electroacupuncture, ischemic stroke, autophagy, PI3K, apoptosis

## Abstract

Electroacupuncture (EA) is a safe and effective therapy for ischemic stroke in both clinical and laboratory settings. However, the underlying mechanism behind EA treatment for stroke remains unclear. Here, we aimed to evaluate whether EA treatment at the acupoints of Zusanli (ST36) and Quchi (LI11) exerted a neuroprotective effect on ischemic stroke rats by modulating autophagy and apoptosis *via* the PI3K/AKT/mTOR signaling pathway. EA was performed at 24 h following brain ischemia/reperfusion (I/R) for 30 min per day for 3 days. Our results indicated that EA treatment significantly decreased neurological deficits and cerebral infarct volume in ischemic stroke rats. Also, EA intervention markedly reduced neuronal apoptosis by suppressing the activation of cleaved caspase-3 (CCAS3) at 72 h following I/R, as shown by a Western blot analysis. Furthermore, EA treatment after ischemic stroke suppressed the ischemia activated expression level of LC3II/I and Atg7 and increased the ischemia inhibited expression level of PI3K, phosphorylation of mTOR, phosphorylation of AKT, P62 and LAMP1, hence mediating the autophagy level of the neurocyte, which was reversed by the PI3K inhibitor Dactolisib. In summary, our results indicate that the protective effects of EA treatment at points of Quchi (LI11) and Zusanli (ST36) in rats following cerebral I/R injury was associated with the inhibition of neuronal apoptosis and autophagy *via* activating the PI3K/AKT/mTOR signaling pathway.

## Introduction

Ischemic stroke is a type of cerebrovascular disease with high morbidity, disability, and mortality; it represents a critical threat to human health and life and is a leading reason for permanent disability and death for adults (Liu et al., 2019; Yang et al., 2019). The pathogenesis of ischemic stroke is complicated. During ischemia/reperfusion (I/R) injury, neuronal cells undergo various acute changes that can result in alterations of various signaling pathways (Xie et al., 2013). As a result of ischemia, the blood supply to neurons is interrupted, which then promotes a series of pathophysiological responses. Various pathological changes are involved individually or jointly in the ischemic process, including autophagy, apoptosis, inflammation, excitatory toxicity, mitochondrial death pathways and free radical release (Khoshnam et al., 2017; Sekerdag et al., 2018), all of which lead to neuronal cell death, hindering important motor (Sun et al., 2012), sensory (Graf et al., 1986) and cognitive functions (Escobar et al., 2019; Liu et al., 2019). Ischemic stroke has been considered one of the main factors that cause aging of the brain and the pathological state of neurodegenerative diseases (Sommer, 2017; Graham and Liu, 2017). However, reliable therapies for ischemic stroke have been very limited until now. Therefore, effective and safe treatments are urgently required.

Comprising the integration of acupuncture and electric stimulation, electroacupuncture (EA) is a safe and effective treatment method in the treatment of various diseases. EA is widely used in experimental research and clinical therapy for ischemic stroke (Liu et al., 2019). According to our previous studies, EA can improve neurological dysfunction, decrease the infarct volumes, and decrease the number of ischemic lesions (Xing et al., 2018a,b). Moreover, EA treatment could generate neuro regenerative effects for ischemic stroke, including promoting brain blood flow, regulating oxidative stress, reducing excitatory amino acids with neurotoxicity, maintaining the integrity of the blood-brain barrier, inhibiting neuronal apoptosis, increasing neurotrophic factors and producing brain ischemic tolerance (Xing et al., 2018c). Therefore, early intervention with EA in the acute stage of ischemic stroke has very important clinical significance.

It is well established that following I/R injury, the brain will undergo more serious damage than with ischemia alone. The mechanisms behind brain I/R damage are complex and associated with neuronal autophagy and apoptosis. Autophagy, which is a key process for cell survival following a stroke, plays a crucial role in the pathogenesis of cerebral ischemia-reperfusion injury (He et al., 2019). Moreover, there is mounting evidence proving that after cerebral I/R, the suppression of apoptosis plays a key role in preventing neuron injury and even death (Zhang et al., 2018).

EA preconditioning can effectively improve cerebral ischemia/reperfusion injury (CIRI) in rats, which may be related to the suppression of autophagy in the ischemic cerebral cortex tissue (Huang et al., 2019). EA preconditioning can reduce the number of autophagosomes and the expression of autophagy markers in the ischemic cortex. Also, both 100 Hz and 2 Hz/10 0HZ-EA are effective in improving the recovery of hindlimb motor function in spinal cord injury (SCI) rats; this may be associated with the function of reducing neuronal apoptosis and promoting the autophagy of damaged nerve cells (Luo et al., 2019). The PI3K/AKT pathway plays an important role in regulating cell proliferation, differentiation, migration, and apoptosis in various types of cells (Samakova et al., 2019). Therefore, the PI3K/AKT pathway is a key target for ischemic stroke treatment. However, whether EA could suppress neuronal autophagy and apoptosis *via* the PI3K/AKT pathway following ischemic stroke is not clear, and the related mechanisms remain unknown.

In the present study, we investigated whether EA treatment at the Quchi (LI11) and Zusanli (ST36) acupoints can provide neuroprotection by regulating autophagy and apoptosis through the PI3K/AKT pathway after ischemic stroke.

## Materials and Methods

### Middle Cerebral Artery Occlusion/Reperfusion (MCAO/R) Model

The MCAO/R animal model was induced by middle cerebral artery (MCA) occlusion. Briefly, each rat fasted in a 12 h light/dark cycle and then anesthetized by intraperitoneal injection of 10% chloral hydrate (300 mg/kg); the left external carotid artery (ECA), left common carotid artery (CCA) and internal carotid artery (ICA) were exposed *via* a midline neck incision. The left MCA was occluded by inserting a surgical nylon suture (diameter, 0.26 mm; Beijing Shandong Biotech Co., Ltd., Beijing, China) through the ICA. After blocking for 2 h, the nylon cord was slowly removed for reperfusion to restore blood supply in the MCA area. This model was measured with the MCAO method, as described previously (Xing et al., 2018a,b). The rectal temperatures of the rats were kept at 37°C throughout the whole surgical process. The rats of the sham-operated group underwent the same surgical procedure without suture insertion. The conditions of occlusions and reperfusion were monitored by laser-doppler flowmetry.

### Animals and Groups

Sprague–Dawley (SD) rats, weighing 250–280 g, were purchased from the Hebei Province Laboratory Animal Center. The SD rats were housed in a 12 h light/dark cycle at a temperature of 22 ± 2°C and 60–70% humidity. Food and water were available *ad libitum*. The experimental protocol was permitted by the Animal Use and Care Committee of Hebei Medical University.

**Figure 1:**
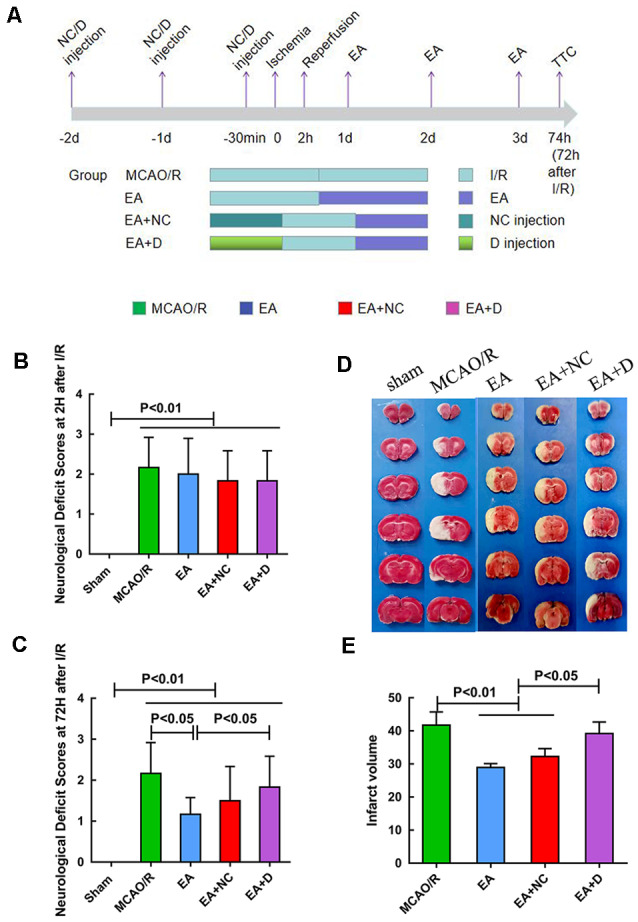
The pathology of an injury following ischemia/reperfusion (I/R) within 3 days. **(A)** Experimental groups and the protocol. **(B)** Neurological deficit assessment at 2 h after I/R injury. **(C)** Neurological deficit assessment at 72 h after I/R injury. **(D)** 2,3,5-triphenyl tetrazolium chloride (TTC) staining for cerebral infarct volume of the sham, MCAO/R, EA, EA+NC, and EA+D groups. **(E)** Bar graph showing the percentage of cerebral infarct volume among the four groups.

As shown in Figure 1A, the 75 rats were randomly divided into five groups (*n* = 15/group) as follows: (i) in the sham group, the rats underwent neck dissection and vascular exposure but no MCA occlusion; (ii) in the MCAO/R group, the left MCA was blocked for 2 h before reperfusion; (iii) in the EA group, the surgical method was the same as that in the MCAO/R group. Reperfusion was performed 2 h after surgery, and EA treatment was administered for 30 min daily for 3 days following MCAO (24, 48, 72 h following ischemia); (iv) in the EA + NC group, NC (the non-specific control of Dactolisib) was provided by intraperitoneal injection daily for 3 days, and the last injection was performed at 30 min before surgery. The rest of the procedures were the same as the EA group; and (v) in the EA+D group, the PI3K inhibitor Dactolisib (Selleck Chemicals, Houston, TX, USA) was dissolved with DMSO, PEG300 and Tween 80 according to the instructions(concentration = 5 mM), which were provided to the rats by intraperitoneal injection daily for 3 days, and the last injection was performed at 30 min before surgery. The other processing methods were the same as those in the EA+NC.

### Assessment of Neurological Deficit Scores

At 2 h and 72 h after I/R, the neurological deficit score was evaluated in a blinded manner: score 0, indicated no neurological deficits; score 1, failure to fully extend right forepaw; score 2, circling to the opposite side; score 3, falling to contralateral side; score 4, not able to walk independently; and score 5, died. Finally, rats with scores of 1–3 points at 2 h following I/R injury were enrolled in the current study.

### EA Intervention

EA intervention was measured with a method previously described. EA intervention was administered at the points Zusanli (ST36) and Quchi (LI11) on the right paralyzed limb *via* an EA apparatus (Model G6805-2A; Shanghai Huayi Co., Shanghai, China). Rats underwent 10% chloral hydrate anesthesia before EA treatment. Two acupuncture needles that were 0.3 mm in diameter were inserted at approximately a 2–3 mm depth at the ST36 and LI11 acupoints. The intervention parameters were set as continuous waves of 2 Hz and an intensity of 1 mA. However, the rats in the sham-operated group and MCAO/R groups were also anesthetized without EA treatment. Finally, the rats were sacrificed at 72 h after MCAO/R for the experiment.

### TTC Staining

The volume of cerebral infarction was detected using TTC staining. Five rats in each group were executed to assess the infarct volume by the TTC method. At 72 h after cerebral I/R injury, the rats were sacrificed under anesthesia with 10% chloral hydrate *via* intraperitoneal injection and stored at −20°C for 15 min. The brain was dissected into six sections in the coronal plane into 2 mm thick slices. The slices were placed in a 2% 2,3,5-triphenyl tetrazolium chloride (TTC, Solarbio, China) solution in 0.1 M phosphate-buffered saline (PBS) at 37°C for 20–30 min and fixed in a 4% paraformaldehyde buffer. The normal brain area was dark red, but no staining was observed in the infarcted area. The infarct was measured using imaging software (Adobe Photoshop 7.0). The formula was used to calculate infarct volume as follows:

Infarct volume = contralateral hemisphere region − a noninfarcted region in the ipsilateral hemisphere.

Infarct percentage=infract volume−volume of the contralateral hemisphere×100%.

### Western Blot Analysis

Western blotting was performed to detect the proteins in the PI3K/AKT/mTOR pathway and autophagy/apoptosis-related proteins. Five rats in each group were executed to detect target proteins. The total proteins were collected from the hippocampal tissue on the left hemisphere of the brain. The hippocampal tissues were homogenized in lysis buffer and centrifuged at 12,000× *g* for 15 min. The supernatants were collected and frozen at −80°C. The protein concentration of each group was detected by the Enhanced BCA Protein Assay Kit (#PC0020, Solarbio, China). Equal amounts of protein (30 μg) were loaded into 12% sodium dodecyl sulfate-polyacrylamide gel electrophoresis (SDS-PAGE) gels for electrophoresis and then transferred onto a PVDF membrane (Roche, Mannheim, Germany). After blocking in a 5% bovine serum albumin solution for 1 h at 37°C, the membranes were incubated at 4°C overnight with primary antibodies against LC3 (1:500, #18725-1-AP, proteintech), P62 (1:1,000, #18420-1-AP, proteintech), LAMP-1 (1:1,000, #62562, Abcam), cleaved-caspase3 (1:500, #66470-2-lg, proteintech), Atg7 (1:1,000, #32345, Cell Signalling Technology, Danvers, MA, USA), PI3K (1:1,000, #4249, Cell Signalling Technology, Danvers, MA, USA), mTOR (1:1,000, #2972 Cell Signalling Technology, Danvers, MA, USA), Phospho-mTOR (1:1,000, #5536, Cell Signalling Technology, Danvers, MA, USA), AKT (1:1,000, #4691, Cell Signalling Technology, Danvers, MA, USA), Phospho-AKT (Thr308, #13038, Cell Signalling Technology, Danvers, MA, USA) and GAPDH (1:5,000, #10494-1-AP, proteintech). After washing with TBST, the membrane was incubated with a goat antimouse IgM (1:5,000) or antirabbit IgG (1:5,000) for 1 h at room temperature. The protein bands were detected using enhanced chemiluminescence (ECL), and the images were analyzed using an Amersham Imager 600. Finally, the optical density of each band was quantified by ImageJ software.

### Immunofluorescence

Rats (*n* = 5/group) were sacrificed without pain. Brain tissue was fixed in formaldehyde, embedded in paraffin, and sectioned with a thickness of 5 mm. Nonspecific antigen binding was blocked with normal sheep serum for 60 min. The sections were separately incubated with primary rabbit antibodies against LC3 (1:200; Cell Signalling Technology, Danvers, MA, USA) and CCAS3 (1:200; Cell Signalling Technology, Danvers, MA, USA) at 4°C overnight. After washing with PBS, the sections were incubated with Rhodamine (TRITC)-conjugated goat antirabbit IgG (H+L) secondary antibody (1:500; proteintech) at room temperature for 1 h in the darks and then washed three times with PBS. Nuclei were counterstained with 4′,6-diamidino-2-phenylindole (DAPI). Cells were observed at 200× magnification under a fluorescence microscope (OLYMPUS 905). Outcomes are shown as optical density (OD). In our present study, immunofluorescence analysis demonstrated the expression difference of LC3 in the ipsilateral hippocampus of the rats in different groups.

### Statistical Analysis

All data were processed using SPSS 21.0. Quantitative data were expressed as the mean ± standard deviation. Differences among the various groups were performed by one-way analysis of variance (ANOVA), followed by a Bonferroni test. The neurological deficits scores among the five groups were analyzed by a nonparametric test. *P* < 0.05 was considered a statistically significant difference.

## Results

### EA Alleviates the Neurological Deficit Scores and Infarct Volumes in Cerebral I/R Injury Rats

The neurological deficit scores were evaluated at 2 h and 72 h after I/R surgery. As shown in Figure 1B, when compared with the sham group, the rats in the other groups exhibited significant neurological deficits (*P* < 0.01), even though there was no significant difference in the neurological deficits score between the MCAO/R group and the EA group at 2 h following I/R. However, EA treatment at the LI11 and ST36 acupoints significantly decreased the neurological deficits score at 72 h following I/R compared with the MCAO/R group (*P* < 0.05), and the neurological deficits of the EA+D group were increased compared with the EA group (*P* < 0.05), as shown in Figure 1C.

### EA Treatment Alleviates Infarct Volumes in Rats following I/R Injury

An infarct volume evaluation was performed at 72 h after I/R injury. As shown in Figure 1D, the rats in the sham group showed no infarct volumes, and there was a significant infarct area in the rest groups. As shown in Figure 1E, the infarct volume of the rats in the MCAO group was significantly higher than the EA group (*P* < 0.01) and EA+NC group (*P* < 0.01). Compared with the EA and EA+NC groups, the infarct volume of the rats in the EA+D group was significantly increased (*P* < 0.05).

### EA Treatment Regulates Autophagy-Related Proteins

**Figure 2:**
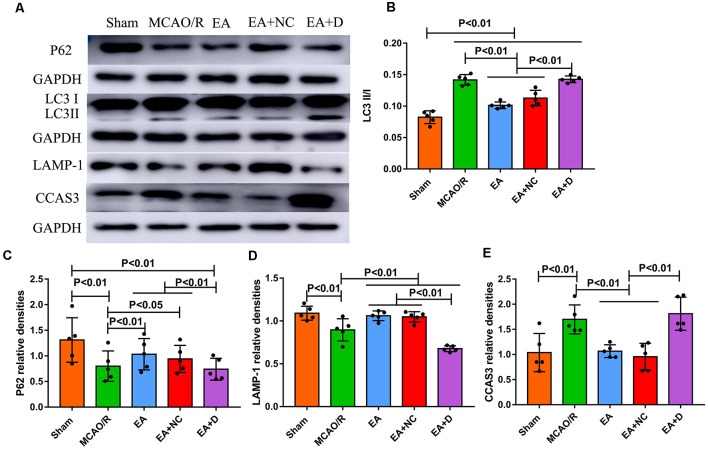
Effect of electroacupuncture (EA) treatment on CCAS3 and autophagy-related proteins. **(A)** Western blot analysis of the expression levels of P62, LC3I, LC3II, LAMP-1, and CCAS3. **(B)** Bar diagram shows the difference of LC3II/I in the ipsilateral hippocampus of the rats (*n* = 5/group). **(C)** Statistical analysis showing the expression of P62 in the ipsilateral hippocampus of the rats. **(D)** Statistical analysis showing the difference of LAMP-1 in the ipsilateral hippocampus of the rats. **(E)** Statistical analysis showing the expression of CCAS3 in the ipsilateral hippocampus of the rats.

The autophagy-related and apoptosis-related proteins were examined by Western blot, including LC3I, LC3II, P62, LAMP-1, and CCAS3, as shown in Figure 2A. As shown in Figure 2B, the ratio of LC3I/II markedly increased in the MCAO/R group compared with the sham group (*P* < 0.01), but EA treatment reversed this change in the EA (*P* < 0.01) and EA+NC groups (*P* < 0.01). Furthermore, the ratio of LC3-I/II in the EA+D group was significantly increased compared with the EA (*P* < 0.01) and EA+NC groups (*P* < 0.01). As shown in Figure 2C, the level of P62 was significantly decreased in the MCAO/R group (*P* < 0.01) compared with the sham group, but EA treatment reversed this change in the EA (*P* < 0.01) and EA+NC groups (*P* < 0.05) compared with the MCAO group, and the expression of P62 in the EA+D group was significantly decreased compared with the EA (*P* < 0.01) and EA+NC groups (*P* < 0.01). Also, as shown in Figure 2D, compared with the sham group, the expression of LAMP-1 in the MCAO/R group was decreased (*P* < 0.01). Moreover, the expression of LAMP-1 was increased in both the EA and EA+NC groups compared with the MCAO group (*P* < 0.01). And the expression of LAMP-1 in the EA+D group was significantly decreased compared with the EA (*P* < 0.01) and EA+NC groups (*P* < 0.01). As shown in Figure 2E, the expression of CCAS3 in the MCAO/R group was significantly increased compared with the sham group (*P* < 0.01). EA intervention at the ST36 and LI11 acupoints significantly decreased neuronal apoptosis *via* reversing the upregulation of CCAS3 in EA (*P* < 0.01) and EA+NC groups (*P* < 0.01). Furthermore, the expression of CCAS3 in the EA+D groups was significantly increased compared with the EA (*P* < 0.01) and EA+NC groups (*P* < 0.01).

**Figure 3:**
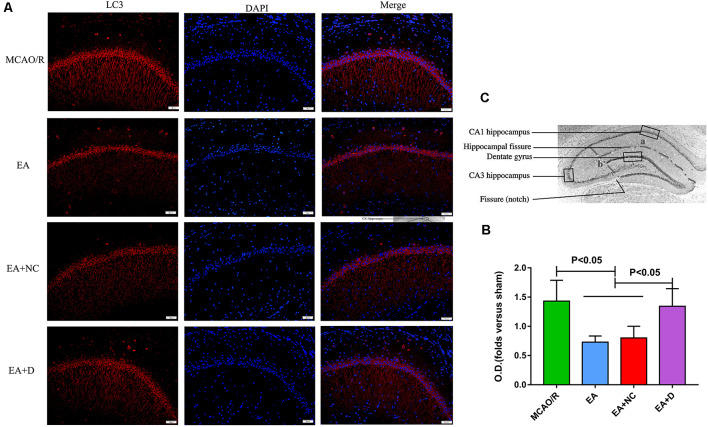
IF analysis of the effect of EA treatment on autophagy-related proteins. **(A)** IF analysis showing the expression difference of LC3 in the ipsilateral hippocampus of the rats. **(B)** Statistical analysis of the LC3 expression assessed by IF. **(C)** The anatomical illustration of the hippocampus in the rat.

To confirm the effect of EA on LC3 expression level, the protein expression of LC3 was evaluated by immunofluorescence, as shown in Figure 3A. As shown in Figure 3B, the LC3 expression level was also significantly decreased in the EA (*P* < 0.05) and EA+NC (*P* < 0.05) groups compared with the MCAO/R group. Comparatively, with the PI3K inhibitor Dactolisib, the level of LC3 in the EA+D group was markedly increased compared with the EA (*P* < 0.05) and EA+NC groups (*P* < 0.05). These results demonstrate that EA treatment after MCAO surgery may inhibit autophagy, which can exert a neuroprotective effect on EA treatment in ischemic stroke. As shown in Figure 3C, the anatomical illustration of the hippocampus in the rat.

### EA Intervention Activates the PI3K/AKT/mTOR Pathway in Cerebral I/R-Injured Rats

**Figure 4:**
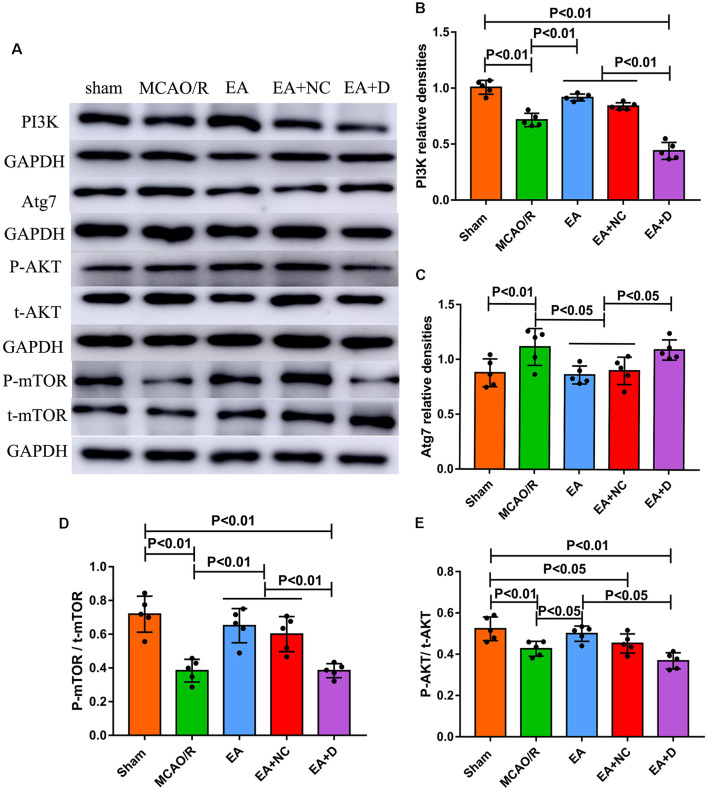
Effect of EA intervention on the protein of the PI3K/AKT pathway in cerebral I/R-injured rats. **(A)** Western blot analysis showing the levels of PI3K, Atg7, P-AKT, t-AKT, P-mTOR, and t-mTOR among the sham, MCAO/R, EA, EA+NC, and EA+D groups. **(B)** Statistical analysis showing the difference of PI3K in the ipsilateral hippocampus of the rats (*n* = 5/group). **(C)** Statistical analysis showing the expression of Atg7 in the ipsilateral hippocampus of the rats. **(D)** Statistical analysis showing the level of P-mTOR/t-mTOR in the ipsilateral hippocampus of the rats. **(E)** Statistical analysis showing the level of P-AKT/t-AKT in the ipsilateral hippocampus of the rats.

As shown in Figure 4A, to investigate the effect of EA intervention on the PI3K/AKT/mTOR pathway following an ischemic stroke, Western blotting was performed to examine the expression levels of PI3K, Atg7, total-mTOR (t-mTOR), phosphorylated-mTOR (P-mTOR), total-AKT (t-AKT) and phosphorylated-AKT (P-AKT) in the ischemic cerebral hippocampus. As shown in Figure 4B, the level of PI3K was significantly decreased in the MCAO/R group (*P* < 0.01) compared with the sham group, but EA treatment reversed this change in the EA (*P* < 0.01) and EA+NC groups (*P* < 0.01). However, the expression of PI3K in the EA+D group was significantly decreased compared with the EA (*P* < 0.01) and EA+NC groups (*P* < 0.01). As shown in Figure 4C, the expression level of Atg7 was increased in the MCAO/R group compared with the sham group (*p* < 0.01). Compared with the MCAO/R group, the expression level of Atg7 was significantly decreased in the EA (*p* < 0.05) and EA+NC groups (*p* < 0.05). Moreover, the expression level of Atg7 was significantly increased in the EA+D group compared with the EA (*p* < 0.05) and EA+NC groups (*p* < 0.05).

As shown in Figure 4D, the levels of phosphorylation of mTOR (P-mTOR/t-mTOR) in the MCAO/R (*P* < 0.01) group were significantly decreased compared with the sham group, whereas EA treatment reversed this change in the EA (*P* < 0.01) and EA+NC groups (*P* < 0.01) compared with the MCAO/R group, and the level of phosphorylation of mTOR in the EA+D group was significantly decreased compared with the EA (*P* < 0.01) and EA+NC groups (*P* < 0.01). The level of phosphorylation of AKT (P-mAkt/t-mAkt) in the MCAO/R group was significantly decreased compared with the sham group (*P* < 0.01). Compared with the MCAO/R group, the level of phosphorylation of AKT was significantly increased in the EA group (*p* < 0.01), but there was no significant difference between the MCAO/R group and EA+NC group. Moreover, the level of phosphorylation of AKT was significantly decreased in the EA+D group compared with the EA group (*p* < 0.01), as shown in Figure 4E. These results demonstrate that EA treatment exerted neuroprotection *via* regulating the PI3K/AKT/mTOR pathway in cerebral I/R rats.

## Discussion

### The Effect of EA Treatment on Autophagy and Apoptosis of Neuronal Cells after Stroke

The protective effect of electroacupuncture on ischemic stroke and other neurological diseases was confirmed by the current study. Our previous studies indicated that EA improved the symptoms of neurological impairment and decreased the volume of cerebral infarction in rats (Xing et al., 2018a,b). However, the exact mechanisms are still not clear. Autophagy plays an important role in the process of cerebral I/R injury and is crucial for neuronal cell survival and death (Harvey, 1979). It was reported that EA preconditioning protected the brain from I/R damage by inhibiting the autophagy process (Wu et al., 2015). Furthermore, EA has been shown to exert a protective effect on ischemic stroke by inhibiting autophagy and autophagosome formation, which is mediated by the mTORC1-ULK complex-Beclin1 pathway (Liu et al., 2016). In the current study, we demonstrated that the expression of P62 and LAMP1 were significantly decreased in the hippocampus after MCAO surgery and were increased in the hippocampus after EA treatment and reversed by the PI3K inhibitor Dactolisib; also, the LC3-II/I ratio was significantly increased after MCAO surgery and was decreased in the hippocampus after EA treatment and was reversed by Dactolisib administration. These results indicate that EA exerted a neuroprotective effect *via* inhibiting neuronal autophagy, which is following the above-mentioned results. Moreover, our results demonstrate that PI3K plays a key role in the process of EA exerting neuroprotection. However, Wu et al. (2016) reported that the protective effect of EA preconditioning for cerebral ischemic injury was mainly because of upregulated autophagy expression. This result was different from our results regarding whether the activation or inhibition of autophagy could play a protective role. The contradictory results might be because of the different time points of EA intervention or the different stages in the pathological process of stroke.

Apoptosis is one important pathophysiological change following an ischemic stroke. Here, a neuroprotective effect is defined as the suppression of neuronal apoptosis to rescue or inhibit the progress of neuronal death (Hou et al., 2018). EA pretreatment can inhibit the expression of proapoptotic genes and proteins, reduce cell apoptosis, and modulate the activation of microglia (Yao et al., 2019). Moreover, EA can alleviate neuronal apoptosis in the ischemic in the penumbral region surrounding the ischemic cores. In the meanwhile, EA activated the expression level of p-AKT, p-Bad, and BCL-2 and inhibited the expression level of Bax and CCAS3 (Xue et al., 2014). In the current study, we found that the expression of CCAS3 was significantly increased after MCAO surgery and was decreased after EA treatment. Moreover, the inhibition of PI3K by Dactolisib abolished the neuroprotective effect of EA. According to these results, EA intervention markedly suppressed neuronal autophagy and apoptosis, thus alleviating brain damage following I/R.

Also, as for acupoint selection, the related animal experiments have shown that electroacupuncture stimulation at LI11 and ST36 acupoints can reduce motor dysfunction, improve the degree of neurological impairment and reduce the volume of cerebral infarction (Xing et al., 2018b,c; Liu et al., 2019). In consideration of the therapeutic dose of EA, based on the results of related articles, we select the 72 h time point and decide on a 3 times*30 min treatment plan in the present study (Liu et al., 2016; Huang et al., 2019).

In summary, EA treatment could exert a neuroprotective effect possibly *via* inhibiting neuronal autophagy and apoptosis following an ischemic stroke, and here, PI3K played a key role in this mechanism.

### The Effect of EA Intervention on PI3K/AKT/mTOR Pathway

Because of the key role of PI3K in the process of inducing neuroprotection *via* EA following an ischemic stroke, the downstream protein targets of PI3K were investigated.

In the present study, the levels of PI3K, phosphorylation of AKT, and phosphorylation of mTOR were significantly decreased following I/R injury, were activated by EA treatment, and were reversed by Dactolisib administration. Moreover, the expression of Atg7 was significantly increased after MCAO surgery, decreased in the hippocampus after EA treatment and reversed by Dactolisib administration. Furthermore, there is substantial evidence that the mammalian targets of rapamycin complex 1 (mTORC1) have an important effect on autophagy after ischemic stroke (Liu et al., 2016). Gabapentin (GBP) pretreatment has been shown to reduce brain IR injury by activating the PI3K/Akt/mTOR pathway, which exerted the neuroprotective effect *via* inhibiting the neurons autophagy associated with oxidative stress (Yan et al., 2019). Moreover, EA treatment could suppress hippocampal neuron apoptosis and improve neurological impairments in rats with cerebral palsy *via* regulating the PI3K/AKT signaling pathway (Zhang et al., 2018). The neuroprotective effect of EA seems to be regulated *via* activating the PI3K pathway rather than the ERK pathway (Sun et al., 2005). Therefore, we could conclude that AKT, mTOR, and Atg7 are the downstream proteins of PI3K.

The PI3K-Akt-mTOR signaling pathway plays an important role in cell autophagy, and it is also a main signal transduction cascade involved in cell proliferation, metabolism, and survival (Wang et al., 2018). LC3, P62, and Beclin-1 are autophagy-related proteins. Beclin-1 interacts with PI3K to trigger autophagy and then participates in the subsequent steps of the fusion of autophagosomes and lysosomes (Zhao et al., 2020). LC3 combines with the autophagosome membrane to form a complete autophagosome, and then fuses and degrades with lysosomes (Currarino et al., 1976). P62 interacts with LC3 II to aggregate for achieving autophagy -specific degradation (Parzych and Klionsky, 2014). The present study demonstrated that EA treatment can activate the PI3K/AKT signaling pathway after ischemic stroke. EA activated the expression level of PI3K, p-AKT, and P-mTOR and inhibited the expression level of Atg7. Moreover, Wang et al. (2019) reported that EA intervention also suppressed the activation of the mTOR signaling pathway and attenuated hermal pain responses in SCI rats. Catalpa may be involved in axonal regeneration *via* regulating the PI3K/AKT/mTOR pathway (Wang et al., 2019). And Rhy (Rhynchophylline) can activate the PI3K/Akt/mTOR signaling pathway possibly by regulating the AKT/mTOR pathway to alleviate ischemic injury (Huang et al., 2014). According to these results, we can conclude that EA treatment exerted neuroprotective effects by regulating the PI3K/AKT/mTOR pathway after ischemic stroke.

## Conclusion

In conclusion, our results indicate that EA at the LI11 and ST36 acupoints inhibited neuronal autophagy and apoptosis in rats following ischemic stroke. Also, the mechanism of EA treatment exerted neuroprotection after ischemic stroke by regulating the PI3K/AKT/mTOR pathway. Furthermore, our research also may provide a possible interventional target point for novel therapeutic methods.

## Data Availability Statement

All datasets generated for this study are included in the article.

## Ethics Statement

The animal study was reviewed and approved by Animal Use and Care Committee of Hebei Medical University.

## Author Contributions

M-MW and FZ designed the study. YX, MZ, and Y-SF performed the experiments. W-BL, Z-XT, FD, M-MW, and FZ analyzed the results together. M-MW and FZ wrote the article. All authors read and approved the final version.

## Conflict of Interest

The authors declare that the research was conducted in the absence of any commercial or financial relationships that could be construed as a potential conflict of interest.
